# Closure of mesenteric defects during Roux-en-Y gastric bypass for obesity: A systematic review and meta-analysis protocol

**DOI:** 10.1016/j.isjp.2019.02.003

**Published:** 2019-02-26

**Authors:** Rhys Thomas, Torsten Olbers, Jonathan D. Barry, Andrew J. Beamish

**Affiliations:** a Department of General Surgery, Abertawe Bro Morgannwg University Health Board, Swansea, UK; b Department of Gastrosurgical Research and Education, Institute of Clinical Sciences, Gothenburg University, Gothenburg, Sweden; c Department of Surgery, Norrköping, Linköping University, Linköping, Sweden; d Welsh Institute of Metabolic and Bariatric Surgery, Swansea, UK

**Keywords:** Meta-analysis, Mesenteric defect, Obesity surgery, Laparoscopic Roux-en-Y gastric bypass surgery, Internal herniation, Small bowel obstruction

## Abstract

**Introduction:**

Closure of mesenteric defects during laparoscopic Roux-en-Y gastric bypass surgery (RYGB) has not been fully established as standard operative practice. However, in recent years a body of evidence has emerged suggesting that non-closure of defects leads to increased rates of internal herniation and its potential consequences, including the need for reoperation, along with an associated morbidity and mortality risk. Within the emerging literature there has also been some evidence of a greater risk of 30-day complications in closure groups. This systematic review and meta-analysis aims to look at the existing evidence and provide guidance on whether closure of mesenteric defects should be standard operative practice.

**Methods:**

The systematic review and meta-analysis has been registered *a priori*. A literature search will be performed interrogating the Medline and Embase databases via Ovid, and also the Cochrane Controlled Register of Trials (CENTRAL), to identify randomised and non-randomised studies reporting comparative outcomes following closure vs. non-closure of mesenteric defects during RYGB. The primary outcome will be reoperation for small bowel obstruction, and secondary outcomes will include internal herniation, jejuno-jejunal anastomosis narrowing or kinking, adhesions, complications (<30 days and >30 days after surgery), 30-day mortality, reoperation, and any other outcome deemed relevant and reported in more than one study.

## Highlights

• Roux-en-Y gastric bypass is an effective treatment for severe obesity and its comorbidities.
• Closure of mesenteric defects may reduce the overall incidence of internal herniation.
• Closure of mesenteric defects may also increase early small bowel obstruction and bleeding.
• This review is designed to establish the evidence for and against closure of mesenteric defects.

## 1 Background

### 1.1 Introduction

Metabolic/bariatric surgery (MBS) has become an important and highly effective treatment in the management of obesity and its related diseases, such as hypertension and type 2 diabetes. A Cochrane review of 22 randomised control trials showed MBS to achieve more effective weight loss after two years than non-surgical measures [1] and this superior efficacy has been shown to endure for at least 2 decades and is accompanied by multiple health improvements [2]. Several types of bariatric procedure exist and laparoscopic Roux-en-Y gastric bypass (RYGB) has remained a popular choice, with particularly impressive endurance and comorbidity resolution [3].

Internal herniation and intermittent internal herniation are recognised late complications of RYGB and represent a common cause for reoperation. Small bowel internal herniation may occur through the retroalimentary space, through the mesojejunal defect created by the jejuno-jejunal anastomosis, or through the transmesocolic space, and can lead to potentially life-threatening small bowel obstruction, ischaemia and necrosis. Several studies have suggested that the routine primary closure of these mesenteric defects may decrease the rates of internal hernia, but this practice is currently not routinely adopted worldwide.

### 1.2 Rationale

Closure of the various mesenteric defects is a relatively simple and quick task to perform, which may reduce risk of internal herniation and the complications thereof. A number of closure methods have been described, including the use of sutures, surgical clips and topical adhesives. If demonstrated to be effective upon consolidation and comparison of all existing data, it may be possible to recommend closure as standard practice worldwide in order to reduce the incidence of these long-term complications. However, it is also essential to document and take account of the extent of excess complications associated with closure of mesenteric defects, such as early reoperation for bleeding and small bowel obstruction. The findings may raise questions regarding the management of patients who have already undergone RYGB without mesenteric defect closure [4].

## 2 Objectives

To identify, retrieve and assess all studies comparing the incidence of early and late complications following closure of mesenteric defects during laparoscopic RYGB surgery, with the primary outcome being reoperation for suspected small bowel obstruction, and secondary outcomes including internal herniation, kinking or narrowing at the jejuno-jejunal anastomosis, adhesions, complications (<30 days and >30 days after surgery), 30-day mortality, and any other outcome deemed relevant and reported in more than one study.

We hypothesise that closure of mesenteric defects: 1. decreases incidence of internal herniation and thus the need for reoperation; 2. is safe to perform, with acceptable rates of immediate and delayed complications.

## 3 Method

This review will follow the recommendations of the Cochrane Handbook for Intervention Reviews (version 5.1), will be compliant with AMSTAR 2 [5] and will be reported in line with the Preferred Reporting Items for Systematic Reviews and Meta-Analyses (PRISMA) statement [6]. This protocol has been developed *a priori*, and the review is registered on the PROSPERO international prospective register of systematic reviews (www.crd.york.ac.uk/prospero); registration number: CRD42018118934.

### 3.1 Study inclusion and exclusion criteria

A summary of inclusion and exclusion criteria is found in Table 1.

**Table 1:** Study inclusion and exclusion criteria.

Inclusion criteria	Exclusion criteria
RCT or comparative observational study	Does not meet inclusion criteria
Roux-en-Y gastric bypass (RYGB)	No comparison group (e.g. only reports closure)
Compares closure of any mesenteric defect with non-closure	Literature review
Operation performed for obesity	Superseded by more recent published work on same patients
Follow-up for at least 30d	Operation not performed for obesity
	Non-English language manuscript

### 3.2 Search strategy

Embase and Medline (via ovid), the Cochrane Central Register of Controlled Trials, and ClinicalTrials.org databases will be searched using the strategy illustrated in Table 2. The search will be restricted to items published in English language up to 1st January 2019. The reference lists of included studies, and the related articles function of Medline via PubMed will be searched for additional relevant studies.

**Table 2:** Embase and Medline (via Ovid) search strategy.

**1. EMBASE and Medline (via Ovid)**
1. gastric bypass.mp. [mp = ti, ab, hw, tn, ot, dm, mf, dv, kw, nm, kf, px, rx, an, ui]
2. gastric bypass*.mp. [mp = ti, ab, hw, tn, ot, dm, mf, dv, kw, nm, kf, px, rx, an, ui]
3. RYGB.mp. [mp = ti, ab, hw, tn, ot, dm, mf, dv, kw, nm, kf, px, rx, an, ui]
4. LRYGB.mp. [mp = ti, ab, hw, tn, ot, dm, mf, dv, kw, nm, kf, px, rx, an, ui]
5. roux*.mp. [mp = ti, ab, hw, tn, ot, dm, mf, dv, kw, nm, kf, px, rx, an, ui]
6. gastrojejunostom*.mp. [mp = ti, ab, hw, tn, ot, dm, mf, dv, kw, nm, kf, px, rx, an, ui]
7. 1 or 2 or 3 or 4 or 5 or 6
8. hernia.mp. [mp = ti, ab, hw, tn, ot, dm, mf, dv, kw, nm, kf, px, rx, an, ui]
9. hernia*.mp. [mp = ti, ab, hw, tn, ot, dm, mf, dv, kw, nm, kf, px, rx, an, ui]
10. mesenteric defect*.mp. [mp = ti, ab, hw, tn, ot, dm, mf, dv, kw, nm, kf, px, rx, an, ui]
11. petersen.mp. [mp = ti, ab, hw, tn, ot, dm, mf, dv, kw, nm, kf, px, rx, an, ui]
12. jejunostomy hernia.mp. [mp = ti, ab, hw, tn, ot, dm, mf, dv, kw, nm, kf, px, rx, an, ui]
13. small bowel obstruction.mp. [mp = ti, ab, hw, tn, ot, dm, mf, dv, kw, nm, kf, px, rx, an, ui]
14. 8 or 9 or 10 or 11 or 12 or 13
15. internal.mp. [mp = ti, ab, hw, tn, ot, dm, mf, dv, kw, nm, kf, px, rx, an, ui]
16. antecol*.mp. [mp = ti, ab, hw, tn, ot, dm, mf, dv, kw, nm, kf, px, rx, an, ui]
17. retrocol*.mp. [mp = ti, ab, hw, tn, ot, dm, mf, dv, kw, nm, kf, px, rx, an, ui]
18. mesenter* closure.mp. [mp = ti, ab, hw, tn, ot, dm, mf, dv, kw, nm, kf, px, rx, an, ui]
19. 15 or 16 or 17 or 18
20. 7 and 14 and 19

### 3.3 Types of studies included

Randomised controlled trials and comparative non-randomised studies comparing closure vs. non-closure of mesenteric defects for RYGB, and reporting outcomes of interest in an extractable form, will be included. Non-comparative studies, review papers, case reports/series, published abstracts, along with reports not written in English will be excluded.

### 3.4 Types of participants

The population of interest is adults (aged 18 years and over) undergoing RYGB for obesity. Studies examining individuals under the age of 18 years will be excluded.

### 3.5 Types of intervention

The intervention of interest will be closure of any or all of: the retroalimentary space, jejuno-jejunal mesenteric defect and/or transmesocolic space during RYGB, using sutures, staples or topical adhesive compounds.

### 3.6 Types of comparator

Only comparative studies will be included and the comparator of interest will be non-closure of any or all of the retroalimentary space, jejuno-jejunal mesenteric defect and/or transmesocolic space during RYGB. Subgroup analyses will be undertaken where data are available, to determine the relative effects of closure of each specific anatomical defect.

### 3.7 Primary outcome

The primary outcome will be reoperation for suspected small bowel obstruction after RYGB.

### 3.8 Secondary outcomes

Secondary outcomes will include internal herniation, kinking or narrowing at the jejuno-jejunal anastomosis, adhesions, complications (<30 days and >30 days after surgery), 30-day mortality, and any other outcome deemed relevant and reported in more than one study. In anticipation of variation in the duration of follow up in relevant studies, a time frame of at least one year of follow-up will be considered as beyond the short term, and subgroup analyses of different follow-up time points will be undertaken where feasible and necessary.

### 3.9 Identification and selection of studies

Articles identified from the electronic search will be recorded into a Microsoft Excel 2017 database with titles and abstract. Duplicates will be excluded. Two separate researchers will independently screen titles and abstracts and code inclusion status as: include (1), exclude (2), or undetermined (3). The full text of all studies coded 1 or 3 will be examined for secondary coding. Discussion between coding researchers will be undertaken, with arbitration by a third author as required, to reach a consensus on final coding for inclusion decisions.

### 3.10 Data extraction, collection and management

Data extraction will be performed by two researchers independently, resolving discrepancies to consensus, with arbitration by a third author where necessary. Data will be input into a Microsoft Excel 2017 database, formatted *a priori* to facilitate simple and consistent data entry.

The following core information will be gathered from each study:

1) Author names, country and year of publication
2) Study design and level of evidence according to Oxford Centre for Evidence-based medicine
3) Number of patients
4) Conflicts of interest and funding
5) Number of participants
6) Age of participants, expressed as mean or median, with standard deviation or range, where reported
7) Method of closing retroalimentary space, jejuno-jejunal mesenteric defect and transmesocolic space
8) Rates of reoperation for suspected small bowel obstruction
9) Rates of internal herniation
10) Rates of kinking or narrowing at the jejuno-jejunal anastomosis
11) Rates of adhesions
12) Rates of complications (<30 days after surgery)
13) Rates of complications (>30 days after surgery)
14) 30-day mortality
15) Immediate post-operative bleeding and leak rates
16) Percentage excess weight loss at 1 year, 3 years and longest follow-up time point
17) Average time to reoperation
18) Follow-up duration

### 3.11 Data analysis

The meta-analysis will be performed in line with the recommendations of the Cochrane Collaboration and the PRISMA guidelines. Analysis will be performed within the Review Manager V.5.3 (RevMan) programme. Statistical analysis will be performed on aggregate data, using proportions for binary outcomes and mean (standard deviation) values for continuous outcomes (or standardised mean values if different scales are used). For outcomes reported in at least five studies, we will fit both random-effects and fixed-effect models. For outcomes reported in less than five studies, we will display the effect estimates in forest plots and perform fixed-effect meta-analysis where appropriate. We will report results with 95 per cent confidence intervals, as well as the I-squared index to assess consistency among results within each outcome.

### 3.12 Assessment of bias

The Cochrane tool will be used in the assessment of randomised studies and the Newcastle-Ottawa scale for non-randomised studies.

## Ethical approval

Ethical approval is not needed for this research project as it does not involve direct contact with patients or direct reporting of identifiable or individual patient level outcome data.

## Funding

No funding has been received for this study.

## Author contribution

AB conceived the study. All authors (RT, TO, JB, and AB contributed to study design, protocol development and writing (NB no data collection or data analysis performed for protocol).

## Conflicts of interest

No conflicts of interest are declared.

## Guarantor

Mr Andrew Beamish.

## Research Registration Number

This study has been registered *a priori* on the PROSPERO international prospective register of systematic reviews (www.crd.york.ac.uk/prospero); registration number: CRD42018118934
